# Dietary Supplements in Public Health: Benefits, Risks, and Emerging Gel/Hydrogel Delivery Systems

**DOI:** 10.3390/gels12030210

**Published:** 2026-03-04

**Authors:** Anca Daniela Raiciu, Georgeta Alexandru, Cristina-Mihaela Luntraru, Mihaela Neagu, Cornelia Manoliu, Cristina Manea, Justinian Andrei Tomescu, Mihaela Carmen Eremia, Amalia Stefaniu

**Affiliations:** 1 Department of Pharmacognosy, Phytochemistry, and Phytotherapy, Faculty of Pharmacy, Titu Maiorescu University, Gh. Şincai Street No. 16, District 4, 040314 Bucharest, Romania; 2 Planta Romanica Association, George Enescu Street No. 27–29, District 1, 010303 Bucharest, Romania; 3 Hofigal Export-Import S.A., Intrarea Serelor Street No. 2, District 4, 042124 Bucharest, Romania; 4 National Institute for Chemical-Pharmaceutical Research and Development, ICCF, 112 Vitan Av. District 3, 031299 Bucharest, Romania; mihaelaceremia@yahoo.com (M.C.E.);

**Keywords:** plant-based dietary supplements, gels, nutraceutical delivery systems, edible hydrogels, bioactive encapsulation, public health nutrition, functional biomaterials

## Abstract

In recent years, the global use of dietary supplements has surged, largely due to their appeal as tools for improving well-being and preventing disease. While these products may offer clear advantages—such as addressing micronutrient shortages and enhancing physical or mental performance—they also carry significant risks, including toxicity, potential drug interactions, and limited clinical validation. This paper explores the current body of scientific literature on dietary supplements, offering a nuanced analysis of their advantages and drawbacks, particularly in general and military populations. Drawing upon peer-reviewed studies, regulatory documents, and expert guidelines, the review outlines key safety considerations and presents practical recommendations for evidence-based use. In addition to conventional formulations, attention is given to the emergence of nutritional gels and hydrogel-based delivery systems, which are increasingly investigated as strategies to improve portability, gastrointestinal tolerability, and bioavailability of bioactive compounds in high-demand civilian and occupational settings. These platforms illustrate a broader shift toward advanced supplement technologies and precision nutrition approaches.

## 1. Introduction

Over the past decade, the dietary supplements sector has undergone robust commercial expansion. In 2023, the global market was estimated at approximately USD 170–180 billion, with projections exceeding USD 250 billion by 2030 and a CAGR of 6–8%, driven by demographic aging, preventive health behaviors, and a shift toward personalized nutrition solutions. The United States, China, and Western Europe account for the largest consumer markets, with vitamins and minerals representing the dominant commercial segment, followed by botanicals, omega-3 fatty acids, probiotics, protein and amino acid formulations, and performance-enhancing supplements. Post-pandemic consumer trends have intensified interest in immune-support ingredients (e.g., vitamin D, zinc, probiotics), cognitive enhancers (“nootropics”), and formulations tailored for sports and high-demand occupational activities [1].

From a regulatory perspective, dietary supplements are concentrated sources of nutrients or bioactive compounds designed to complement the normal diet. In the U.S., the Dietary Supplement Health and Education Act (DSHEA, 1994) categorizes supplements as food-like products that do not require pre-market authorization [2]. Within the European Union, supplements fall under food legislation with variable national interpretations, leading to heterogeneous regulatory frameworks and classification systems. Commercially, dietary supplements encompass several major categories, including vitamins and minerals; amino acids and proteins; probiotics and prebiotics; omega-3 and polyunsaturated fatty acids; and botanicals or phytochemicals. Consumer motivations for supplement use differ across populations, ranging from dietary correction and micronutrient adequacy to performance enhancement, disease prevention, and cognitive or immune support.

Supplement consumption is particularly prevalent among older adults, athletes, and individuals with dietary restrictions. More recently, military and high-performance occupational groups have adopted supplements to sustain physical endurance, focus, and stress resilience under challenging operational conditions. The growing integration of supplements into everyday health routines has also been facilitated by direct-to-consumer marketing, digital health platforms, and the mainstreaming of “preventive” self-care paradigms.

Despite their popularity, the scientific consensus remains divided—especially when these supplements are used without medical guidance. In certain demographics, such as older adults, athletes, or individuals with dietary restrictions, supplements may serve as valuable tools for maintaining nutritional adequacy and physiological resilience. Similarly, within military populations, they are often used to sustain energy levels, concentration, and overall performance under demanding conditions [1,3,4,5].

Nevertheless, concerns persist regarding improper use, excessive intake, inconsistencies in labeling, and interactions with pharmaceuticals [6,7].

Adding to the complexity, the level of regulatory oversight differs widely between countries, leading to variable product quality and safety standards. This review seeks to critically examine the current state of knowledge surrounding dietary supplements, with a focus on both their therapeutic potential and their limitations. Special attention is given to clinical relevance, occupational health considerations, and the importance of responsible consumption practices, especially in environments with heightened physical and psychological stress.

While traditional dietary supplement reviews primarily focus on clinical efficacy and public health impact, increasing attention is now directed toward material-driven delivery strategies. In this context, gel and hydrogel matrices are not only alternative dosage forms but functional biomaterials capable of modulating bioactive stability, release kinetics, and gastrointestinal performance.

Therefore, this review emphasizes nutraceutical gel and hydrogel systems from a structure–property–function perspective, linking material composition and network architecture to biological performance and translational feasibility.

The present review focuses primarily on gel and hydrogel delivery systems designed for nutraceutical and dietary bioactive compounds. Pharmaceutical hydrogel applications are discussed only when mechanistically relevant for understanding material design principles applicable to oral or functional food systems.

To provide a structured overview, this review first summarizes the public health relevance, benefits, and risks associated with dietary supplement use. It then discusses emerging semi-solid nutritional gel formulations used in high-demand physiological contexts, followed by an analysis of hydrogel-based delivery systems as advanced platforms for improving the stability, bioavailability, and targeted delivery of bioactive compounds.

### 1.1. Addressing Micronutrient Deficiencies

The potential benefits of dietary supplements can be broadly categorized into three main domains: correction of micronutrient deficiencies, enhancement of physical and cognitive performance, and supportive roles in clinical care.

Dietary supplements are often essential in preventing or correcting micronutrient imbalances, especially among individuals with increased nutritional needs or limited dietary variety. Deficiencies in elements such as vitamin D, iron, and vitamin B12 remain prevalent in various populations, particularly among the elderly, vegans, or those with chronic illnesses [1,8].

### 1.2. Improving Physical and Mental Performance

A variety of supplements have been investigated for their potential to enhance performance in both physical and cognitive domains. Substances like creatine, caffeine, and branched-chain amino acids (BCAAs) are widely used by athletes and active individuals to support muscle recovery, strength gains, and exercise endurance (Kreider et al., 2010) [9]. On the cognitive side, omega-3 fatty acids, particularly EPA and DHA, have been associated with improved memory, concentration, and overall brain health [10].

### 1.3. Adjunctive Role in Clinical Care

Beyond general wellness, certain supplements have demonstrated promise in clinical contexts. For example, probiotics have been studied for their beneficial effects on gut microbiota and gastrointestinal disorders. Similarly, antioxidants such as coenzyme Q10 and vitamins C and E have shown potential in reducing oxidative damage in patients with cardiovascular conditions or metabolic disorders [11].

### 1.4. Risks and Limitations

Taking high doses of certain vitamins or minerals—especially fat-soluble ones like vitamins A, D, E, and K—can lead to toxic effects. Symptoms range from mild digestive discomfort to serious complications involving liver or kidney damage. Over-the-counter availability of supplements often leads to unsupervised consumption, increasing the likelihood of overdose or misuse.

In contrast to pharmaceuticals, dietary supplements are often subject to less stringent oversight. Research has exposed inconsistencies between label claims and the actual contents of products, as well as the presence of harmful or unlisted ingredients [6].

Although some supplements are supported by scientific research, many others are promoted without strong clinical evidence [9]. Products marketed with vague promises or exaggerated claims frequently underdeliver in real-world use.

Dietary supplements can alter the way drugs are absorbed or metabolized in the body. For instance, vitamin K can reduce the efficacy of anticoagulants, while magnesium might interfere with certain antibiotics [7].

### 1.5. Perspective and Occupational Health

Members of the armed forces often face intense physical exertion, sleep deprivation, and psychological stress. In such conditions, dietary supplements may offer perceived or real benefits in maintaining energy, focus, and endurance. Nonetheless, the unregulated nature of some supplements and their potential for containing banned substances make them a liability. Military health systems are increasingly encouraging evidence-based use and close supervision of supplement intake to mitigate health risks and preserve operational readiness [3].

### 1.6. Structure–Property–Function Relationships in Nutraceutical Gels

The performance of nutraceutical gel systems is governed by the interplay between polymer structure, crosslink density, water content, and mesh size distribution. These parameters directly influence diffusion-controlled release, protection of encapsulated bioactives, and interaction with gastrointestinal fluids.

For example, highly crosslinked networks typically provide improved protection against oxidation and enzymatic degradation but may limit release rate, while loosely crosslinked matrices enable faster nutrient availability but lower stability during storage and digestion.

### 1.7. Gel-Based Nutraceutical Delivery Systems

While traditional dietary supplements are typically delivered as tablets, capsules, or powders, recent advances in nutritional science and material engineering have enabled the development of alternative delivery formats designed to improve portability, gastrointestinal tolerance, and nutrient bioavailability.

In addition to traditional supplement formats such as capsules, tablets, and powders, recent developments have introduced semi-solid nutritional gels as portable, rapidly absorbed sources of carbohydrates, electrolytes, amino acids, and micronutrients. These formulations are widely used in endurance sports and physically demanding settings where fast energy delivery, minimal gastrointestinal burden, and operational convenience are essential.

Beyond nutritional gels designed primarily for rapid energy delivery, hydrogel-based systems represent a more advanced technological approach, focusing on controlled release, protection of sensitive bioactive compounds, and targeted delivery at the tissue or cellular level.

Parallel to these trends, hydrogel-based systems have emerged as advanced delivery platforms capable of encapsulating natural bioactive compounds, improving stability, solubility, and targeted release. Together, nutritional gels and hydrogel matrices reflect a broader shift toward innovative supplement technologies and precision nutrition strategies [12,13,14,15,16,17,18,19,20,21,22,23,24,25,26,27,28,29,30,31,32,33,34,35].

While these aspects provide essential public health context, the present review specifically focuses on gel and hydrogel delivery systems as material-driven nutraceutical technologies. Conceptually, nutraceutical gel and hydrogel systems can be viewed as an interface between public health nutrition and biomaterial-enabled delivery science. In these systems, formulation architecture directly influences bioactive stability, dosing precision, and physiological availability. This framework allows integration of population-level nutritional needs with material-level delivery optimization, supporting the development of precision nutraceutical strategies adapted to specific physiological and occupational demands.

## 2. Regulatory, Technological and Translational Perspectives

### 2.1. Regulatory Frameworks for Dietary Supplements in the U.S. and EU

This section discusses dietary supplements from regulatory, technological, and application perspectives, beginning with general regulatory frameworks and progressing toward emerging gel-based and hydrogel-based delivery technologies. Regulatory approaches to dietary supplements differ widely across countries. In the U.S., the Food and Drug Administration treats supplements more like food than medicine, limiting pre-market evaluation. Meanwhile, the European Food Safety Authority provides structured but inconsistently enforced guidelines. Harmonizing global standards and introducing mandatory quality controls could improve product consistency and safety [2].

While supplements can play a beneficial role in personal and public health, misuse and poor oversight can result in significant harm. Aggressive marketing and easy access contribute to their widespread, and sometimes inappropriate, consumption. More robust, long-term studies are necessary to better understand their effects, especially in high-risk or performance-demanding populations.

This paper explores the current legal and regulatory framework surrounding traditional plant-based food supplements and herbal medicinal products within the European Union (EU) and USA. The sale of botanical ingredients in foods and dietary supplements is governed by various EU food laws, which address issues such as safety, manufacturing standards, labeling, and product formulation—including rules on additives and limits for contaminants and residues. However, because EU-wide harmonization in this area remains incomplete, individual Member States have also implemented their own national rules. As a result, products can be marketed across different EU countries through the principle of mutual recognition, even if regulations differ [36,37,38,39].

In contrast, traditional herbal medicinal products are subject to a specific directive—Directive 2004/24/EC—which thoroughly outlines the regulatory requirements, including a simplified national registration process. By drawing a line between traditional herbal medicines and plant-based food supplements and introducing separate pathways for their authorization, the EU has encountered both challenges in enforcement and a lack of consistency in regulatory practices among Member States.

Currently, the classification and availability of botanical products—whether marketed as traditional medicines or as supplements—depend heavily on the specific interpretations and regulatory decisions of national authorities and producers in each country. This leads to considerable variation across the EU, not necessarily based on the properties of the botanical ingredients themselves, but on how rules are applied. When compared to regulatory models in other parts of the world, significant differences become apparent in how these types of products are handled.

### 2.2. Regulatory Considerations for Nutritional Gels and Hydrogel-Based Delivery Systems

Nutritional gels occupy an intermediate regulatory position, as they are formulated as food-derived matrices delivering carbohydrates, electrolytes, amino acids, and other nutrients in semi-solid form. In both the United States and the European Union, such products are generally regulated under food or dietary supplement legislation, with classification depending primarily on composition, claims, and intended use. Within endurance sports and military settings, nutritional gels are commercialized as dietary supplements aimed at providing rapid energy replenishment and improved gastrointestinal tolerability under prolonged physical exertion [3,9,40,41,42].

**Figure 1:**
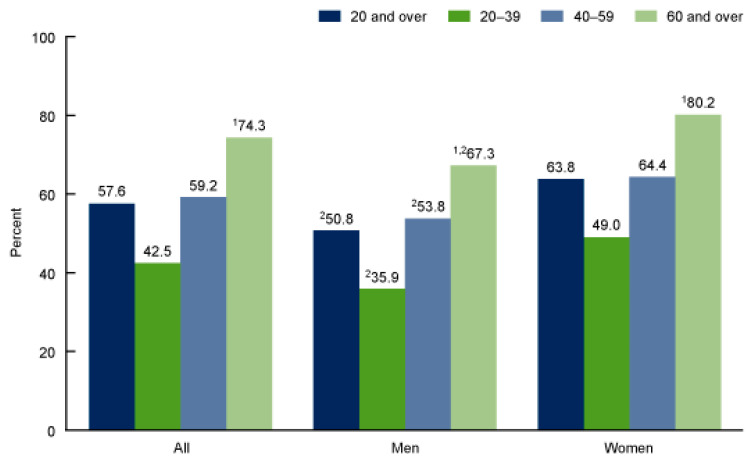
Percentage of adults aged 20 and over who used any dietary supplement, by sex and age: United States, 2017–2018 [43]. ^1^ Significant linear increasing trend with age. ^2^ Significantly different from women of the same age group.

**Figure 2:**
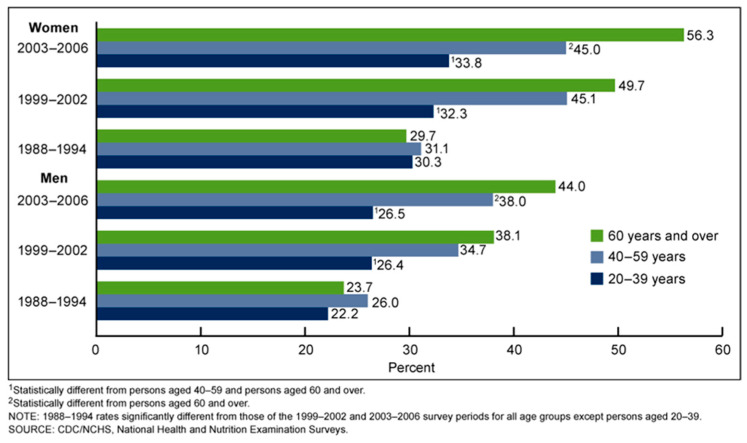
Prevalence of supplemental vitamin D use in adults aged 20 and over, by age group: United States, 1988–2006 [8].

Hydrogel-based systems introduce further complexity from a regulatory standpoint. These three-dimensional polymer networks are increasingly used to encapsulate natural bioactive compounds—including polyphenols, flavonoids, essential oil constituents, and ginsenosides—to enhance their stability, solubility, and controlled release profiles [12,13,14,15,16,17,18,19,20,21,22,23,24,25,26,27,28,29,30,31,32,33,34,35]. Depending on application, hydrogels may fall under dietary supplement, cosmetic, or medicinal product pathways, with national regulatory authorities determining classification based on intended function and therapeutic claims. This heterogeneity highlights the need for clearer regulatory criteria that distinguish between traditional supplements and advanced delivery platforms as biomaterials, functional foods, and nutraceutical technologies continue to converge (Figure 1 and Figure 2).

### 2.3. Gel-Based Nutraceutical Delivery Systems

Nutritional gels represent a specialized category of dietary supplements increasingly used in endurance sports and military environments due to their rapid absorption and ease of administration under physically demanding conditions. These formulations typically contain fast-acting carbohydrates—such as maltodextrin, glucose, or fructose—often combined with electrolytes, amino acids, or bioactive compounds like caffeine, enabling rapid replenishment of muscle glycogen and supporting thermoregulation during prolonged exertion [40,41]. Research demonstrates that carbohydrate gels consumed at regular intervals help maintain blood glucose concentrations, delay fatigue onset, and improve endurance performance, particularly in long-duration activities or operational contexts involving heat stress or load carriage [42].

Despite these benefits, their use requires careful individualization. Excessive intake or inappropriate timing may lead to gastrointestinal discomfort, osmotic diarrhea, or altered glycemic responses, which can impair performance rather than enhance it [44]. Interactions with other supplements—especially caffeinated products or concentrated electrolyte solutions—may further increase risks such as dehydration or cardiovascular strain if not properly supervised. Quality assurance remains a key concern, as variability in ingredient purity, undeclared stimulants, and inconsistencies between labeling and content have been documented in certain commercial gel formulations, posing potential risks for athletes and military personnel subject to strict safety or anti-doping regulations [45].

**Figure 3:**
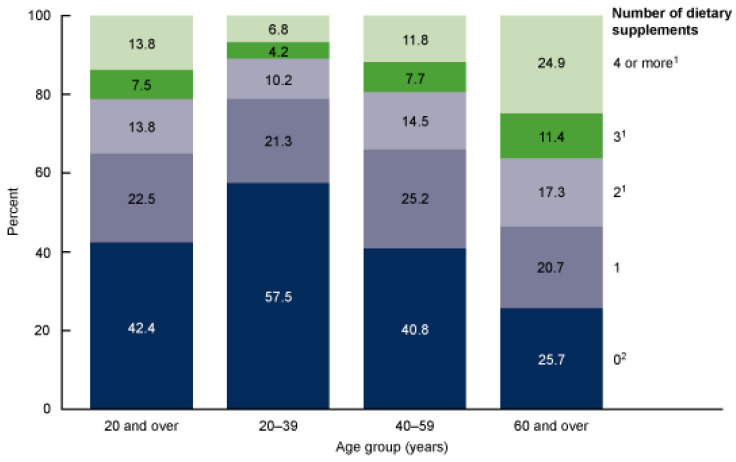
Number of dietary supplements used by adults aged ≥ 20 years, by age group: United States, 2017–2018. The figure illustrates age-related patterns in the number of supplements consumed, showing the tendency toward increased multi-supplement use with advancing age [43]. ^1^ Significant linear increasing trend with age. ^2^ Significantly different from women of the same age group.

**Figure 4:**
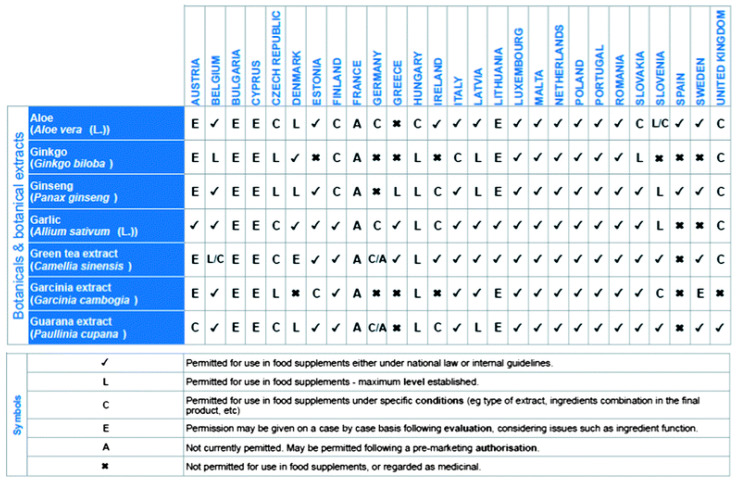
Examples of different national approaches for the use of selected botanicals in food supplements in the Member States of the EU [36].

**Figure 5:**
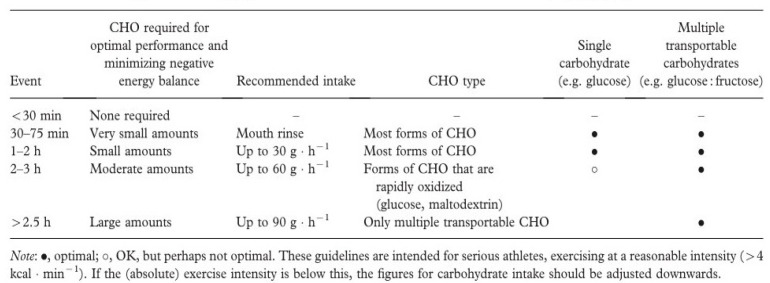
Recommendations for carbohydrate (CHO) intake during different endurance events [40].

**Figure 6:**
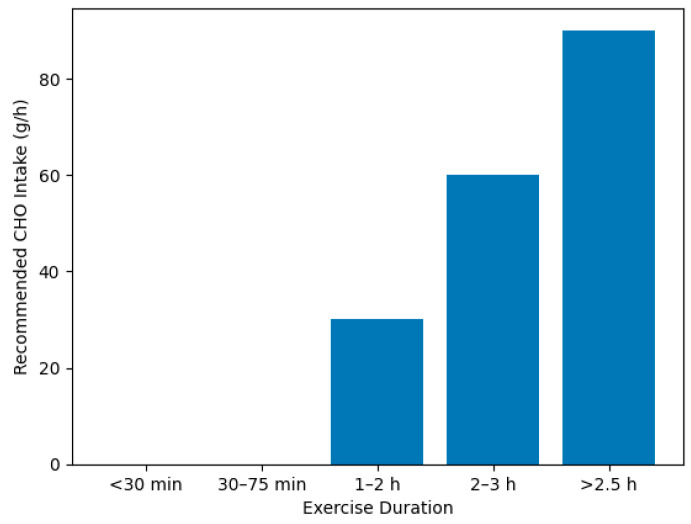
Recommended carbohydrate (CHO) intake during endurance exercise depending on exercise duration. The bar chart illustrates guideline-based carbohydrate intake requirements (g·h^−1^) needed to maintain performance and energy balance during prolonged physical activity. For exercise durations under 30 min, carbohydrate intake is generally not required. During exercise lasting 30–75 min, carbohydrate mouth rinse strategies may be used without significant ingestion. For longer durations, recommended intake progressively increases, reaching approximately 30 g·h^−1^ for 1–2 h, 60 g·h^−1^ for 2–3 h, and up to 90 g·h^−1^ for prolonged endurance exercise exceeding 2.5 h, particularly when multiple transportable carbohydrates are used.

When used according to evidence-based guidelines and within structured nutritional strategies, energy gels can serve as effective adjuncts for sustaining physical performance, cognitive alertness, and resilience during extended operational or athletic challenges (Figure 3, Figure 4, Figure 5 and Figure 6).

### 2.4. Hydrogel Natural Compounds in Therapy

**Figure 7:**
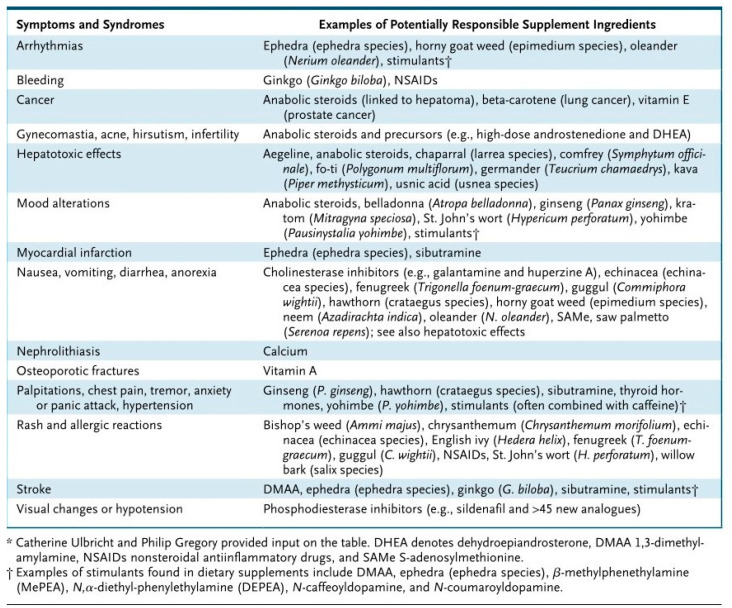
Examples of Potential Adverse Reactions to Legal Ingredients and Adulterants in Dietary Supplements [6].

Natural products derived from renewable and sustainable biological resources continue to serve as invaluable sources of structurally diverse bioactive compounds and secondary metabolites. These molecules—including polyphenols, flavonoids, terpenoids, alkaloids, and polysaccharides—exhibit significant therapeutic potential across antimicrobial, antioxidant, anti-inflammatory, and anticancer applications. However, their translation into clinical or cosmeceutical use is often restricted by physicochemical challenges such as poor water solubility, rapid enzymatic degradation, and limited membrane permeability (Figure 7).

Recent pharmaceutical innovations have prompted the development of advanced hydrogel and nano-hydrogel platforms, which address many of these delivery limitations. Hydrogels—three-dimensional, crosslinked polymeric networks—create a hydrophilic microenvironment ideal for the encapsulation, stabilization, and controlled release of natural compounds. Examples of commonly used hydrogel materials include chitosan, alginate, hyaluronic acid, gelatin, polyvinyl alcohol (PVA), and polyethylene glycol (PEG). These biopolymers are valued for their biodegradability, mucoadhesiveness, and inherent biocompatibility, all of which improve their suitability for pharmaceutical and dermatological applications [12,13,14].

**Figure 8:**
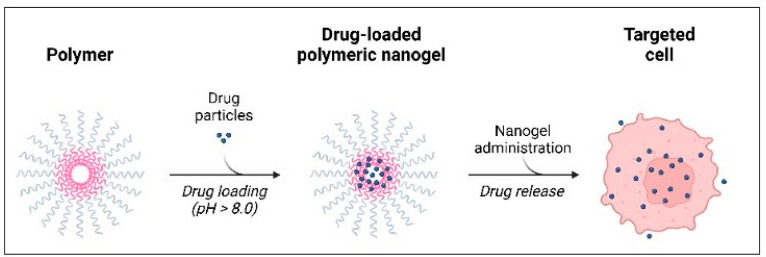
Drug incorporation into nano-hydrogel [12].

Edible hydrogel nutraceutical systems introduce additional regulatory complexity because classification is often claim-dependent. Products positioned for general nutritional support may fall under dietary supplement or functional food legislation, whereas systems making therapeutic or disease-related claims may be regulated as medicinal products. In the European Union, edible hydrogel systems containing novel encapsulation technologies or previously unconsumed polymer matrices may require Novel Food authorization through EFSA evaluation. In the United States, regulatory pathways may involve GRAS (Generally Recognized As Safe) status for polymer matrices combined with dietary supplement regulatory frameworks. These regulatory boundaries directly influence material selection, allowable crosslinking chemistries, acceptable excipient profiles, and long-term safety testing requirements. As edible hydrogel delivery technologies advance, clearer regulatory guidance will be necessary to support safe translation from experimental formulations to consumer-ready nutraceutical products (Figure 8).

### 2.5. Examples of Hydrogels and the Natural Compounds Encapsulated

Although several hydrogel systems originate from pharmaceutical research, they provide mechanistic insight into polymer–bioactive interactions that are directly transferable to nutraceutical and edible gel design.

The following examples illustrate how hydrogel-based systems can enhance the stability, solubility, and therapeutic potential of selected natural bioactive compounds.

### 2.6. Curcumin—Chitosan and PVA Nano-Hydrogels

**Figure 9:**
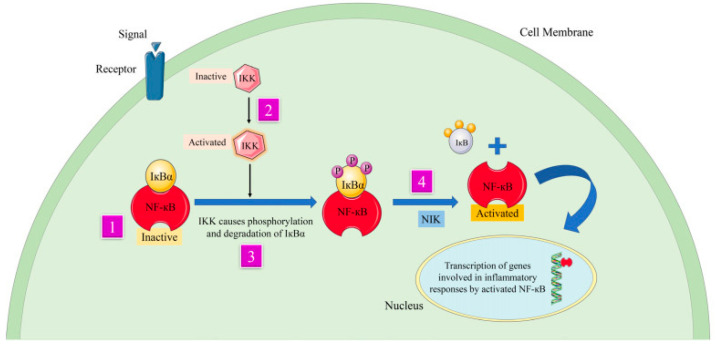
Curcumin deals with the mechanism of inflammation and signaling pathways by suppressing transcription factor NFκB. The numbers 1, 2, 3 and 4 in the figure indicate different pathways involved in the activity of the bioactive compound that makes up curcumin [16].

Curcumin, a hydrophobic polyphenolic compound from *Curcuma longa*, exhibits potent anti-inflammatory and antioxidant effects but suffers from poor aqueous solubility and rapid metabolic degradation. Encapsulating curcumin in chitosan nano-hydrogels enhances mucoadhesion, promotes interaction with negatively charged cellular membranes, and increases epithelial permeability. Chitosan’s pH-responsive swelling further supports controlled release in acidic environments. PVA-based nano-hydrogels improve curcumin’s dispersion and photostability while providing sustained release kinetics, making them suitable for oral, transdermal, and wound-healing applications [15,16] (Figure 9).

### 2.7. Resveratrol—Alginate and Hyaluronic Acid Hydrogels

**Figure 10:**
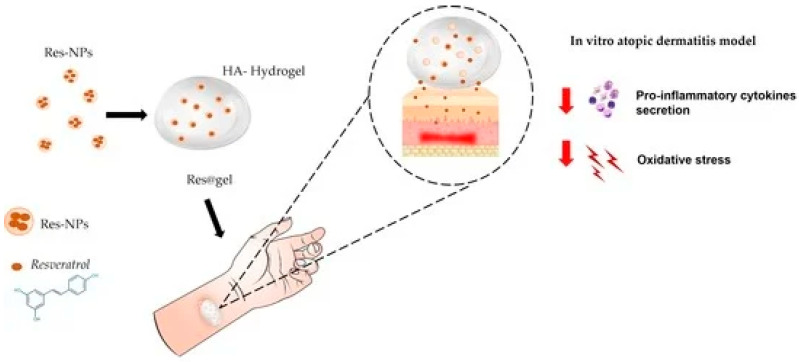
Hyaluronic acid hydrogel containing resveratrol-loaded chitosan nanoparticles as an adjuvant in atopic dermatitis treatment [17].

Resveratrol, a stilbene with well-documented antiaging and cardioprotective properties, is chemically unstable and rapidly oxidized. Alginate hydrogels, crosslinked with divalent cations such as Ca^2+^, protect resveratrol from oxidative degradation and allow mild encapsulation conditions that preserve its structural integrity. Hyaluronic acid (HA) hydrogels, widely used in dermatology, offer excellent hydration and biocompatibility, enhancing dermal penetration of resveratrol and enabling its use in cosmeceutical antiaging formulations [17,18] (Figure 10).

### 2.8. Thymol and Carvacrol—PEG Nano-Hydrogels

**Figure 11:**
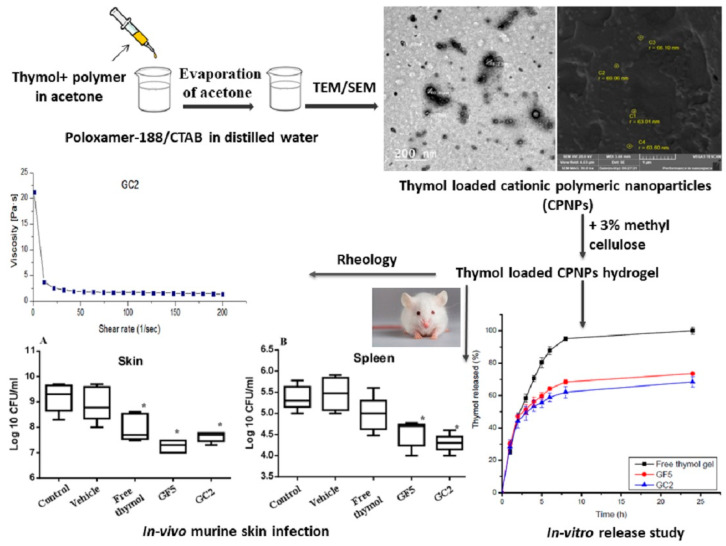
Thymol-loaded Eudragit RS30D cationic nanoparticles-based hydrogels for topical application in wounds: in vitro and in vivo evaluation (A) representation of in vivo analysis of the skin (B) representation of in vivo analysis of the spleen [23].

Thymol and carvacrol, monoterpenoid phenols found in thyme and oregano oils, are potent antimicrobial agents but are volatile and hydrophobic. Encapsulation within PEG-based nano-hydrogels improves their solubility, reduces volatilization, and enables sustained antimicrobial activity. PEG provides a nonionic, biocompatible matrix that stabilizes these essential-oil constituents, enhancing their efficiency against bacterial and fungal pathogens while mitigating irritation associated with direct application [19,20,21,22,23,24] (Figure 11).

### 2.9. Quercetin—Gelatin–Chitosan Hydrogels

**Figure 12:**
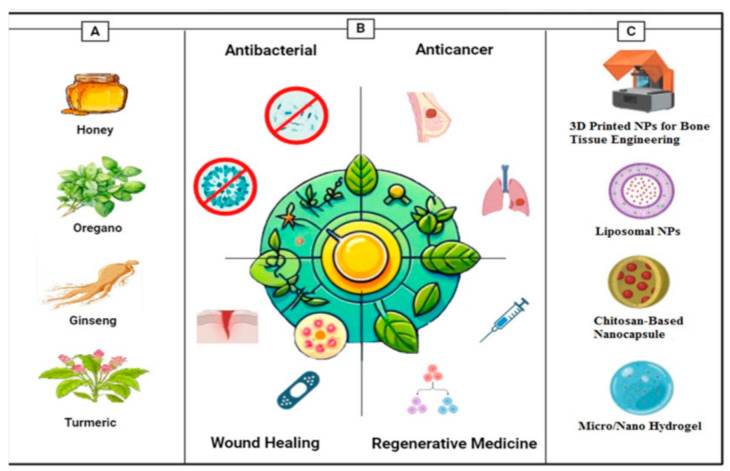
Overall view of this report. (**A**) Commonly used plants and natural compounds and their bioactive compounds [Honey–Turmeric–Ginseng–Oregano]. (**B**) Main applications [antibacterial–anticancer–wound healing–regenerative medicine]. (**C**) Main delivery systems [25].

Quercetin is a flavonoid with strong antioxidant, anti-inflammatory, and cytoprotective properties but has poor water solubility and limited permeability. Gelatin–chitosan composite hydrogels combine the biocompatibility and cell-adhesive properties of gelatin with the mucoadhesive and pH-responsive characteristics of chitosan. This dual-polymer network effectively encapsulates quercetin, increases its aqueous stability, and enhances its transdermal or mucosal absorption. Such systems are promising for wound healing and anti-inflammatory therapies [25] (Figure 12).

### 2.10. Aloe vera Polysaccharides—Natural Hydrogels for Wound Healing

**Figure 13:**
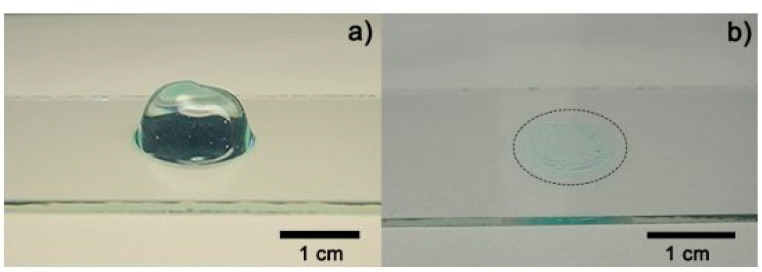
Optical images of *Aloe vera* hydrogel: (**a**) sample before drying process and (**b**) dried (xerogel) [30].

Polysaccharides extracted from *Aloe vera*—including acemannan—possess intrinsic wound-healing, immunomodulatory, and moisturizing effects. When used to form natural hydrogels, these polysaccharides create a moist wound environment, promote fibroblast proliferation, and stimulate collagen synthesis. Their intrinsic bioactivity allows Aloe-based hydrogels to serve as both the matrix and the therapeutic agent, reducing the need for additional actives and supporting applications in burns, ulcers, and tissue regeneration [26,27,28,29,30] (Figure 13).

### 2.11. Ginsenosides—Hyaluronic Acid (HA) Hydrogels

Ginsenosides, the key bioactive compounds of *Panax ginseng*, exhibit antioxidant, antitumor, and antiaging activities but are prone to degradation in biological environments. HA hydrogels provide a hydrated, viscoelastic matrix that protects ginsenosides from enzymatic breakdown and enhances their uptake by skin and tumor tissues due to HA’s natural affinity for CD44 receptors overexpressed in many cancer cells. This receptor-mediated targeting mechanism makes HA–ginsenoside hydrogels attractive for dermatological therapy and localized anticancer delivery [31,32,33,34,35].

Collectively, these examples demonstrate the versatility of hydrogel systems in addressing major delivery limitations associated with natural compounds, including instability, poor solubility, and low bioavailability.

Compared with alternative polymer matrices, this system demonstrates advantages including improved mucoadhesion, hydration capacity, and mechanical stability, but limitations remain regarding large-scale manufacturing reproducibility and long-term physicochemical stability under variable storage conditions.

From a nutraceutical perspective, this system illustrates design principles rather than representing a currently approved dietary supplement formulation.

From an evidence-based perspective, currently marketed nutraceutical gel systems—particularly carbohydrate-based endurance gels—are supported by controlled clinical and performance data. In contrast, most nano-hydrogel encapsulation systems designed for phytochemicals, probiotics, or plant-derived bioactives remain at preclinical or early translational development stages.

**Figure 14:**
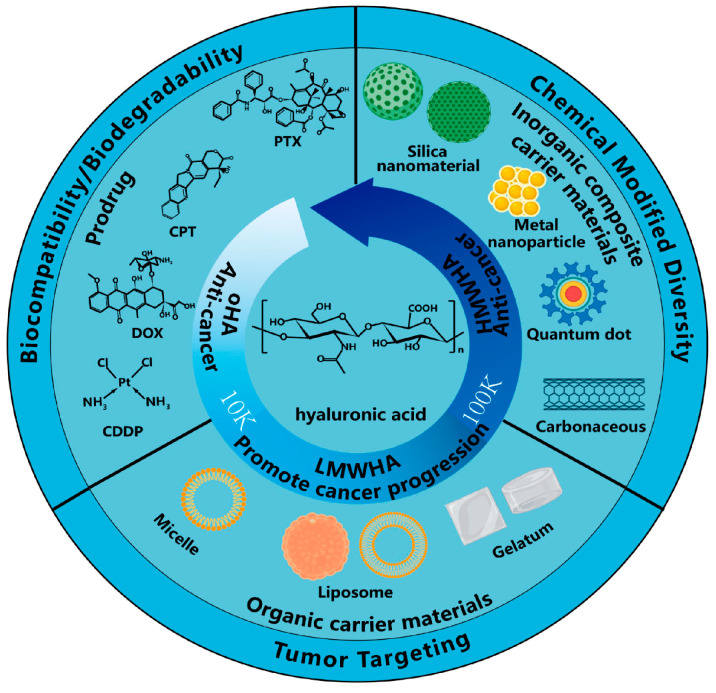
Chemical structure of HA and the hypothesized procancer (promoting cancer growth) and anticancer (preventing cancer growth) activity of HA with different molecular weights and its application [31].

This discrepancy highlights an important translational gap between material innovation and real-world nutraceutical implementation. Future research should prioritize controlled human intervention studies evaluating dosing precision, long-term safety, gastrointestinal tolerance, and interactions with complex dietary matrices. In addition, regulatory-grade toxicological and stability studies will be essential before large-scale commercialization of advanced hydrogel-based nutraceutical systems can be achieved (Figure 14).

### 2.12. Mechanistic Basis for Improved Delivery

Hydrophilic polymer matrices may enhance the apparent solubility of lipophilic compounds through multiple mechanisms, including amorphous stabilization, molecular-level dispersion within the polymer network, or formation of nanostructured domains depending on polymer–bioactive interactions.

Hydrogels enhance the bioavailability of natural molecules through several interconnected mechanisms:

1. Encapsulation and protection
  - Prevents oxidation and enzymatic degradation.
  - Stabilizes sensitive molecules (e.g., curcumin against light and pH instability).
2. Controlled and sustained release
  - Crosslink density governs water diffusion and molecule mobility, enabling release kinetics ranging from hours to weeks.
  - pH-responsive hydrogels (e.g., chitosan swelling at acidic pH) release compounds specifically at target tissues such as gastric or inflamed environments.
3. Enhanced cellular uptake

Nano-hydrogels (<200 nm) facilitate receptor-mediated endocytosis or direct membrane translocation, improving intracellular delivery of hydrophobic compounds such as limonene, thymol, and curcuminoids.

4. Improved solubility and dispersion

Hydrophilic polymer matrices increase the apparent solubility of lipophilic molecules by generating nano-dispersed or colloidal forms.

5. Targeting capabilities (in advanced nano-hydrogels)

Surface functionalization with ligands (e.g., folate, peptides) enables selective delivery to cancer cells or inflamed tissues.

Hydrophobic or hydrophilic natural compounds are trapped within the porous hydrogel network, protected from degradation. Importantly, regulatory classification strongly influences material selection. Food-grade gel systems must rely on biopolymers such as alginate, pectin, gelatin, or modified starch, whereas pharmaceutical hydrogels may incorporate synthetic polymers or more complex crosslinking chemistries. Consequently, regulatory frameworks indirectly shape gel architecture, degradation behavior, and allowable encapsulation strategies.

Despite promising laboratory results, several translational barriers remain. These include batch-to-batch variability in natural polymer sources, scale-up challenges in crosslinking control, stability during long-term storage, and regulatory uncertainty for hybrid nutraceutical–biomaterial systems.

Addressing these challenges will require integration of material science, food engineering, and regulatory science to ensure reproducibility and safety at industrial scale.

## 3. Conclusions

Nutraceutical gel systems represent a convergence point between food science, biomaterials engineering, and personalized nutrition, positioning soft-matter delivery platforms as a key enabling technology for next-generation dietary interventions.

When used judiciously and under professional guidance, dietary supplements can support nutritional adequacy, cognitive and physical performance, and overall health [1,9,10]. These benefits are most substantial when integrated into balanced lifestyle practices and aligned with evidence-based clinical recommendations. Conversely, unsupervised or performance-driven intake—particularly in military and athletic environments—raises concerns related to toxicity, drug–supplement interactions, and overreliance on products that may obscure the importance of proper diet and medical care [3,6,7,45]. Strengthened public education, rigorous quality control, and clearer clinical guidelines are therefore essential to promote responsible use and minimize harm in both civilian and specialized operational settings.

Beyond traditional dosage forms such as tablets, capsules, and powders, nutritional gels have emerged as semi-solid supplement formats that provide concentrated sources of carbohydrates, electrolytes, amino acids, and micronutrients [40,41,42]. Their functional advantages—rapid gastric emptying, ease of ingestion, and low gastrointestinal burden—make them suitable for prolonged endurance activities and high-stress conditions where rapid energy delivery and hydration are required [3,9,40]. In military and emergency-response scenarios, nutritional gels facilitate maintenance of energy balance, electrolyte homeostasis, and cognitive alertness when conventional meals are impractical [3].

Parallel to these developments, hydrogel-based delivery systems represent a more advanced technological frontier. Hydrogels are three-dimensional polymer networks capable of encapsulating bioactive compounds, protecting them from degradation, and releasing them in a controlled or targeted manner [12,13,14,15,16,17,18,19,20,21,22,23,24,25,26,27,28,29,30,31,32,33,34,35]. Such platforms are particularly attractive for sensitive molecules—including antioxidants, probiotics, omega-3 fatty acids, and phytochemicals—whose functional stability may be compromised by heat, oxidation, or gastric acidity [12,13,14,15,16,17,18,25]. Moreover, hydrogel matrices enable modulation of glycemic responses and sustained nutrient release, an advantage in prolonged physical exertion and environments demanding cognitive continuity [40,41].

Material and food engineering advances have further enabled the development of edible hydrogels based on alginate, pectin, agar, carrageenan, gelatin, and modified starch, with applications spanning nutraceutical, dermatological, wound-healing, and regenerative medicine fields [12,13,14,15,16,17,18,19,20,21,22,23,24,25,26,27,28,29,30,31,32,33,34,35]. Emerging hydrogel-based hydration systems illustrate additional use cases, encapsulating water and electrolytes in semi-solid form to improve fluid retention and reduce dehydration risks in austere environments such as deserts, high altitudes, or disaster-relief contexts [40,41,42].

Despite their promise, gel and hydrogel supplement technologies introduce new considerations related to dosing strategies, safety validation, nutritional interactions, and user education. Their regulatory classification remains heterogeneous, ranging from dietary supplement to cosmetic or medicinal product pathways depending on claims, bioactive content, and intended function [2,36]. As these delivery platforms expand, regulatory agencies will need to adapt evaluation criteria to address release kinetics, material biodegradability, and the stability of encapsulated nutrients—challenges not typically encountered with conventional supplement formats.

Clinically, the integration of nutritional gels and hydrogel-based systems into personalized nutrition protocols opens possibilities for populations with dysphagia, gastrointestinal sensitivities, elevated metabolic demands, or performance requirements. However, optimization of these interventions will require standardized research methodologies, robust safety data, and interdisciplinary collaboration between healthcare providers, nutrition scientists, performance specialists, and biomaterial engineers [3,9,12,13,14,15,16,17,18,19,20,21,22,23,24,25,26,27,28,29,30,31,32,33,34,35].

Overall, the rise of gel- and hydrogel-based delivery systems reflects a broader shift toward precision nutrition—approaches that are portable, physiologically adaptive, and technologically sophisticated. When anchored in scientific validation, regulatory oversight, and professional guidance, these platforms hold substantial potential to enhance resilience, health, and performance across civilian, athletic, and military domains [3,9].

Future development of nutraceutical gels will likely rely on rational material design approaches integrating polymer chemistry, microstructure engineering, and predictive release modeling. Such strategies will support the transition from empirical formulation toward precision nutraceutical delivery platforms.

### 3.1. Technologies Ready for Application

Conventional nutraceutical gels, particularly carbohydrate–electrolyte formulations used in endurance sports and high-demand occupational settings, are already supported by clinical performance data and established regulatory frameworks.

### 3.2. Technologies at Experimental or Early Translational Stage

Advanced nano-hydrogel systems designed for phytochemical, probiotic, or multifunctional bioactive delivery remain largely at experimental or early translational stages, with limited clinical validation currently available.

### 3.3. Priority Areas for Future Research and Regulation

Future work should focus on long-term safety assessment, standardized characterization of release kinetics in physiological environments, regulatory harmonization for edible hydrogel biomaterials, and development of clinically validated dosing strategies for complex bioactive delivery systems.

Overall, nutraceutical gel technologies represent a transition from conventional supplement delivery toward rationally engineered soft-matter nutritional platforms, supporting the broader evolution toward precision nutrition and personalized dietary interventions.

However, translation of advanced nano-hydrogel nutraceutical systems into clinically validated or commercially available products remains limited.

## Data Availability

The data that support the findings of this study are available on request from the corresponding author.

## References

[1] Bailey R.L., Gahche J.J., Lentino C.V., Dwyer J.T., Engel J.S., Thomas P.R., Betz J.M., Sempos C.T., Picciano M.F. (2011). Dietary supplement use in the United States 2003–2006. J. Nutr..

[2] Wallace T.C., Koturbash I. (2025). DSHEA 1994–Celebrating 30 Years of Dietary Supplement Regulation in the United State. J. Diet. Suppl..

[3] Austin K.G., Price L.L., McGraw S.M., Lieberman H.R. (2015). Predictors of Dietary supplement use by the U.S. Coast Guard Personnel. PLoS ONE.

[4] Kantor E.D., Rehm C.D., Du M., White E., Giovannucci E.L. (2016). Trends in dietary supplement use among US adults from 1999–2012. JAMA.

[5] NIH Office of Dietary Supplements Vitamin D Fact Sheet for Health Professionals. https://ods.od.nih.gov.

[6] Cohen P.A. (2014). Hazards of hindsight—Monitoring the safety of nutritional supplements. N. Engl. J. Med..

[7] Sood A., Sood R., Brinker F.J., Mann R., Loehrer L.L., Wahner-Roedler D.L. (2008). Potential interactions between dietary supplements and prescription medications. Am. J. Med..

[8] Gahche J. (2011). Dietary Supplement Use Among US Adults Has Increased Since NHANES III (1988–1994).

[9] Kreider R.B., Wilborn C.D., Taylor L., Campbell B., Almada A.L., Collins R., Cooke M., Earnest C.P., Greenwood M., Kalman D.S. (2010). ISSN Exercise & Sport Nutrition Review: Research & recommendations. J. Int. Soc. Sports Nutr..

[10] Yurko-Mauro K., Alexander D.D., Van Elswyk M.E. (2015). Docosahexaenoic acid and adult memory: A systematic review and meta-analysis. PLoS ONE.

[11] Littarru G.P., Tiano L. (2005). Clinical aspects of coenzyme Q_10_: An update. Curr. Opin. Clin. Nutr. Metab. Care.

[12] Muslim M.R.F., Chabib L., Hamzah H. (2025). Nano-hydrogel systems in herbal medicine: A systematic review. J. Herbmed Pharmacol..

[13] Guo X., Luo W., Wu L., Zhang L., Chen Y., Li T., Li H., Zhang W., Liu Y., Zheng J. (2024). Natural products from herbal medicine self-assemble into advanced bioactive materials. Adv. Sci..

[14] Segneanu A.-E., Bejenaru L.E., Bejenaru C., Blendea A., Mogoşanu G.D., Biţă A., Boia E.R. (2025). Advancements in Hydrogels: A Comprehensive Review of Natural and Synthetic Innovations for Biomedical Applications. Polymers.

[15] Kenawy E.-R.S., Kamoun E.A., Ghaly Z.S., Shokr A.-B.M., El-Meligy M.A., Mahmoud Y.A.-G. (2023). Physically cross-linked curcumin-loaded PVA/*Aloe vera* hydrogel membranes for acceleration of topical wound healing: In vitro and in vivo experiments. Arab. J. Sci. Eng..

[16] Chopra H., Bibi S., Mohanta Y.K., Mohanta T.K., Kumar S., Singh I., Khan M.S., Rauta P.R., Alshammari A., Alharbi M. (2023). In vitro and in Silico characterization of curcumin-loaded chitosan–PVA hydrogels: Antimicrobial and potential wound healing activity. Gels.

[17] Conte R., De Luca I., Valentino A., Cerruti P., Pedram P., Cabrera-Barjas G., Moeini A., Calarco A. (2023). Hyaluronic acid hydrogel containing resveratrol-loaded chitosan nanoparticles as an adjuvant in atopic dermatitis treatment. J. Funct. Biomater..

[18] Law S.K., Liu C.W.C., Tong C.W.S., Au D.C.T. (2024). Potential of resveratrol to combine with hydrogel for photodynamic therapy against bacteria and cancer—A review. Biomedicines.

[19] Folle C., Díaz-Garrido N., Mallandrich M., Suñer-Carbó J., Sánchez-López E., Halbaut L., Marqués A.M., Espina M., Badia J., Baldoma L. (2024). Hydrogel of Thyme-Oil-PLGA Nanoparticles Designed for Skin Inflammation Treatment. Gels.

[20] Moghtaderi M., Bazzazan S., Sorourian G., Sorourian M., Akhavanzanjani Y., Noorbazargan H., Ren Q. (2003). Encapsulation of Thymol in Gelatin Methacryloyl (GelMa)-Based Nanoniosome Enables Enhanced Antibiofilm Activity and Wound Healing. Pharmaceutics.

[21] Yammine J., Chihib N.-E., Gharsallaoui A., Ismail A., Karam L. (2023). Advances in essential oils encapsulation: Development, characterization and release mechanisms. Polym. Bull..

[22] Naz S., Javaid S., Rehman S.U., Razzaq H. (2025). Recent advances in polymer nanoencapsulation of essential oils for biomedical and industrial applications. Mater. Adv..

[23] Mohsen A.M., Nagy Y.I., Shehabeldine A.M., Okba M.M. (2022). Thymol-Loaded Eudragit RS30D Cationic Nanoparticles-Based Hydrogels for Topical Application in Wounds: In Vitro and In Vivo Evaluation. Pharmaceutics.

[24] Fakhariha M., Rafati A.A., Garmakhany A.D., Asl A.Z. (2025). Nanoencapsulation enhances stability, release behavior, and antimicrobial properties of Sage and Thyme essential oils. Sci. Rep..

[25] Najafi F., Farrokhzad N., Ghaemi A., Khezri D.A., Hajiabbas M., Khanizadeh A., Farsani N.K., Khoramipour M., Fatemipayam N., Zadeh E.S. (2025). Advancement in nanocarrier-mediated delivery of herbal bioactives: From bench to beside. Nat. Prod. Bioprospect..

[26] Chithra P., Sajithlal G.B., Chandrakasan G. (1998). Influence of *Aloe vera* on collagen turnover in healing of dermal wounds in rats. Indian J. Exp. Biol..

[27] Bai Y., Niu Y., Qin S., Ma G. (2023). A new biomaterial derived from *Aloe vera*—Acemannan from basic studies to clinical application. Pharmaceutics.

[28] Eshun K., He Q. (2004). *Aloe vera*: A valuable ingredient for the food, pharmaceutical, and cosmetic industries—A review. Crit. Rev. Food Sci. Nutr..

[29] Sánchez M., González-Burgos E., Iglesias I., Gómez-Serranillos M.P. (2020). Pharmacological Update Properties of *Aloe vera* and its Major Active Constituents. Molecules.

[30] Meza-Valle K.Z., Saucedo-Acuña R.A., Tovar-Carrillo K.L., Cuevas-González J.C., Zaragoza-Contreras E.A., Melgoza-Lozano J. (2021). Characterization and Topical Study of *Aloe vera* Hydrogel on Wound-Healing Process. Polymers.

[31] Fu C.-P., Cai X.-Y., Chen S.-L., Yu H.-W., Fang Y., Feng X.-C., Zhang L.-M., Li C.-Y. (2023). Hyaluronic acid-based nanocarriers for anticancer drug delivery. Polymers.

[32] Kim J.H., Moon M.J., Kim D.Y., Heo S.H., Jeong Y.Y. (2018). Hyaluronic Acid-Based Nanomaterials for Cancer Therapy. Polymers.

[33] Nag S.A., Qin J.J., Wang W., Wang M.H., Wang H., Zhang R. (2012). Ginsenosides as Anticancer Agents: In vitro and in vivo Activities, Structure–Activity Relationships, and Molecular Mechanisms of Action. Front. Pharmacol..

[34] He B., Chen D., Zhang X., Yang R., Yang Y., Chen P., Shen Z. (2022). Oxidative Stress and Ginsenosides: An Update on the Molecular Mechanisms. Oxidative Med. Cell. Longev..

[35] Ju Y., Hu Y., Yang P., Xie X., Fang B. (2023). Extracellular vesicle-loaded hydrogels for tissue repair and regeneration. Mater. Today Bio.

[36] Silano V., Coppens P., Larrañaga-Guetaria A., Minghetti P., Roth-Ehrang R. (2011). Regulations applicable to plant food supplements and related products in the European Union. Food Funct..

[37] EFSA (2012). Compendium of botanicals. EFSA J..

[38] Teschke R., Andrade R.J. (2016). Drug, Herb, and Dietary Supplement Hepatotoxicity. Int. J. Mol. Sci..

[39] Reflection Paper on the Use of Information in European Union Herbal Monographs and Assessment Reports for Borderline Issues. https://www.ema.europa.eu/system/files/documents/scientific-guideline/reflection-paper-use-information-eu-herbal-monographs-assessment-reports_en_0.pdf.

[40] Jeukendrup A.E. (2011). Nutrition for endurance sports: Marathon, triathlon, and road cycling. J. Sports Sci..

[41] Cermak N.M., van Loon L.J.C. (2013). The use of carbohydrates during exercise as an ergogenic aid. Sports Med..

[42] Pfeiffer B., Stellingwerff T., Zaltas E., Jeukendrup A.E. (2010). CHO Oxidation from a CHO Gel Compared with a Drink during Exercise. Am. Coll. Sports Med..

[43] Mishra S., Stierman B., Gahche J.J., Potischman N. (2021). Dietary Supplement Use Among Adults: United States 2017–2018.

[44] Rosado-Dawid N.Z., Torres-León J.M., Portales-Núñez M.E., Ramos-Meca M.A., García-Mayor M.Á., de-la-Torre-Gutiérrez S. (2013). Ischemic colitis due to vigorous physical exercise. Rev. Esp. Enferm. Dig..

[45] Martirosyan D., Tavva S. (2025). The science, safety, and policy of dietary supplements: A comprehensive review and future roadmap. Diet. Suppl. Nutraceuticals.

